# Challenges and added value of measuring embodied variables in psychotherapy

**DOI:** 10.3389/fpsyt.2022.1058507

**Published:** 2022-12-16

**Authors:** Petra Nyman-Salonen, Virpi-Liisa Kykyri, Markku Penttonen

**Affiliations:** ^1^Department of Psychology, Faculty of Education and Psychology, University of Jyväskylä, Jyväskylä, Finland; ^2^Department of Social Sciences and Philosophy, Faculty of Humanities and Social Sciences, University of Jyväskylä, Jyväskylä, Finland

**Keywords:** embodiment, psychotherapy, synchrony, nonverbal synchrony, electrodermal activity, methodology, crossmodal, embodied variables

## Abstract

Research on embodied aspects of clinical encounters is growing, but discussion on the premises of including embodied variables in empirical research is scarce. Studies have repeatedly demonstrated that embodied aspects of psychotherapy interaction are vital in developing a therapeutic alliance, and these should be considered to better understand the change process in psychotherapy. However, the field is still debating which methods should be used and which features of the embodied aspects are relevant in the clinical context. The field lacks methodological consistency as well as a theoretical model. In the Relational Mind research project, we have studied the embodied aspects of interaction in the context of couple therapy for almost a decade and have gained experience with the positive and negative aspects of studying embodied variables in quantitative and qualitative studies. We have set out to develop the methodology (or procedures) for studying embodied variables in a multiperson setting, concentrating on interpersonal synchrony of sympathetic nervous system responses and movements, and we have strived to create methods for integrating information from different embodied modalities. In this narrative review, we share our experiences of the challenges and added value of studying embodied aspects in psychotherapy. The research field urgently needs an ongoing discussion of what researchers should take into consideration when studying the embodied aspects of interaction. We urge researchers to collaborate between research groups to jointly decide on the basic parameters of studies on the different embodied modalities of the research so that the individual researcher can become more aware of the impact the methodological choices have on their studies, results, and interpretations. We also see the use of embodied variables as having added value in the clinical work of psychotherapists, since it not only deepens our understanding about what is helpful in psychotherapy but will enable fine-tuning therapy processes to better suit clients who are verbally less fluent.

## Introduction

Recently, there has been growing interest in studying the embodied aspects of psychotherapy to investigate how they are related to the change process of psychotherapy and its outcome. Embodied aspects have been suggested as one of the common factors (1) shared among psychotherapy approaches that account for the effectiveness of the treatment (2, 3).

Research has concentrated on the nonverbal aspects of communication in clinical contexts (4, 5), such as patients' autonomic activation during clinical interaction [for a review, see (6)], blood pressure during suppressed emotions (7), how the client's nonverbal behavior during clinical interactions can be related to diagnostics (8), and how general practitioners and psychiatrists differ in their embodied implicit mentalization strategies (9). In this paper, we claim that research on embodied factors in the clinical context is still in its creative exploratory phase, during which researchers try out different methods and variables. To develop the research field further, we as researchers need to be aware of how our methodological choices, e.g., the operationalizations, the choice of data analysis units, and analysis methods impact the results of our studies, and how the context impacts the meaning and possible interpretations that can be made from the results. We will use our experience from the research project *Relational Mind in Events of Change in Multiactor Therapeutic Dialogues* (10, 11) to illustrate the difficulties researchers face when studying embodied variables, and what aspects a researcher needs to take into account while designing the study, collecting the data, and analyzing and interpreting the results. This article is a narrative review of research conducted in this area and aims to enhance readers' understanding of using embodied variables in clinical research.

## Research on embodiment

In research in the psychotherapeutic context, studies on embodiment have mainly been conducted using three research approaches: (i) Qualitative studies on the verbal dialogue in psychotherapy sessions have added embodied aspects of the interaction to the discourse analysis (12–14); (ii) quantitative studies using larger sample sizes have extracted specific embodied variables from psychotherapy sessions, such as movement energy by the participants, and calculated synchrony between the client and therapist based on these (15–17); and (iii) autonomic reactions, such as skin conductance, heart rate, breathing, or movements of the participants in the therapy sessions, have been recorded using wearable trackers [cf. (18–22)]. The study of interpersonal synchrony is a growing field of research. In psychotherapy, synchrony between client and therapist has been found in several modalities: in movements (15, 16, 23), in physiological variables, such as skin conductance (21, 24, 25), heart rate and heart rate variability (26), and even respiration (18). Although research on the embodied aspects of psychotherapy or other clinical contexts has been increasing, articles discussing the challenges and added value of studying embodied variables in psychotherapy are lacking. The aim of this narrative review is to examine different aspects of conducting research on embodied variables in psychotherapy.

This paper is inspired by our experience from the Relational Mind research project (10), in which we have conducted research on the embodied aspects of couple therapy interactions. It is important to stress, that we have not made experimental research, but gathered data from natural interactions in couple therapy.

We will first present the Relational Mind research project and the research field which studies embodied variables in natural settings. After that we will dwell into different aspects that need to be considered when conducting research on embodied variables, such as how to gather, process and analyze data originating from different modalities of interaction. After this we address the complexity of studying embodied data, its idiosyncrasy and context dependent nature. Relevant issues regarding synchrony studies are addressed as well, since we have in the Relational Mind research project studied synchrony between participants in different embodied modalities. Relevant issues regarding synchrony calculations and inferences made based on the findings are addressed.

We have also strived to integrate information from different modalities and will disclose our research strategies on how this could be done, with a hope to develop the research field further. Finally, we will discuss the added values of using embodied variables, as well as make some recommendations for researchers in the field. We have in our studies used several different methods but find that discussion about methodology is scarce. We hope that this paper will start a scientific discussion on the premises of studying embodied variables in psychotherapy.

## The Relational Mind research project

The Relational Mind research project (2013–present) conducted at the Psychotherapy Training and Research Centre of the Department of Psychology at the University of Jyväskylä has focused on embodied aspects and especially interactional synchrony in couple therapy (10, 11). The premise of the Relational Mind (hereafter RM) project was to study how the participants' mutual attunement is formed by clients and therapists in couple therapy. The multimodal design, which uses couple therapy as a “natural laboratory,” provides access to verbal, nonverbal, physiological, and experiential levels of therapy interactions. The rationale and the design of the study has been described in detail in Seikkula et al. (10, 11), and the findings of the study have been reported in several empirical publications (all RM studies are listed in the Appendix). The RM design was novel and innovative in 2013, and even today, it is rare in psychotherapy process research. In RM couple therapy was conducted in a usual manner, but the sessions were video-recorded and the participants' (both clients' and therapists') autonomic nervous system responses (electrodermal activity, heart rate, and respiration) were recorded in two measurement sessions, one at the beginning of the therapy process and the other toward the end of the process. After the measurement sessions, individual stimulated recall interviews (hereafter SRIs) were conducted, for which the researcher had chosen four episodes from the therapy session, and the participants were asked to recall what thoughts, feelings, and bodily sensations they recall having at that time in the session. The data comprise 12 couple therapy processes, including 150 h of therapy sessions video-recorded with six cameras, 23 sessions during which clients' and therapists' psychophysiological responses were measured, and 92 individual video-assisted interviews. Moreover, in each session, alliance and outcome measures were used. Detailed information on the research design and participants can be found in Karvonen (27), and for an interested reader the theoretical background of the design is described in Seikkula et al. (11).

To analyze the data, a wide range of methodological approaches and tools, including qualitative conversational and observational approaches as well as mixed methods and computational and statistical approaches, were used. The embodied side of the interaction was included in the analysis of all the studies.

We discovered that even though research on embodied aspects is vital to obtaining a more complete picture of what occurs during clinical interactions, this type of research has proven to be very complex. The reason for this is at least 3-fold: (i) There is no preexisting common methodology for studying the embodied aspects of psychotherapy interventions. Studies use different methods, which is seen through the entire research process: in the variety of research designs, in the differences in selecting the data analysis unit, and the differences in interpreting the results. This makes the research field scattered and hinders the comparison of the studies. We found that we needed to separately define or develop (or tailor-make) a data analysis method for each study that could best answer the research questions. (ii) The use of different methods is related to the lack of a common theory that could connect the findings from different studies on embodiment in clinical encounters. Embodied cognition [cf. (28)], which sees all cognitive processes to be grounded in the body's interactions with the world, has been suggested as one of the theories, as well as complexity science [cf. (29–31)], but these are still too abstract to be easily applicable to studies in clinical contexts. (iii) The embodied variables often prove to be context dependent, with the meaning of the same behavior (such as synchrony) changing from one situation to the other. Embodied variables are often idiosyncratic, meaning that, for instance, people use their bodies in individual ways, and embodied aspects influence persons in idiosyncratic ways, which leads to difficulty in interpreting the results. This was found in our study on nonverbal synchrony in couple therapy, where we discovered that the associations between, for instance, synchrony and the alliance changed depending on the role of the participant (client or therapist) or even gender (23). This aspect has been most profoundly manifested in qualitative RM studies that have concentrated on shorter episodes of the dialogue combining embodied variables into the analysis, which have demonstrated that the participants interpret the situations individually (32, 33). These aspects make it difficult to compare different studies. Thus, insufficient information has accumulated for us to form a common theory.

As we tried to grasp the various aspects of embodied research, it became important to consider the development of the research field in its historical context. We compared research on embodied variables to brain research, which has a long tradition of methodological development, starting from feature extraction (e.g., figuring out what signals were relevant) to considerations of how the information obtained should be processed to obtain the key aspects of the signal in relation to the research question. When inspecting research on embodied aspects, it became evident that, in this research area, we are still in the early phases of research, trying to figure out which features are relevant and what kind of feature detection methods we should use. This phase of research has taken almost a century and is still an ongoing process. One reason for this is that the term *embodied* is an umbrella term covering many aspects of interaction, from visible features such as nonverbal behavior to invisible behavior such as electrodermal activity, heart rate, and breathing frequency. Due to this, the research field is very large, and the various embodied variables require the development of methods to extract and process them. To make research even more complex, the variables of the different modalities can be used or operationalized in various ways, ranging from specific nonverbal behaviors such as head nods (34) to head movement synchrony (35) or psychophysiological arousal (36) to synchrony of electrodermal activity (EDA synchrony) (37). While meta-analyses of research on only visible nonverbal behaviors in the clinical context have repeatedly concluded that it is important to study these behaviors but that research has not enhanced much in recent decades (38, 39), it is not surprising that the vast field of studying embodied variables suffers from similar difficulties.

Based on these observations, we suggest that the field is still in its creative exploratory phase, during which we try different methods to extract features such as EDA synchrony [for an excellent discussion in this regard to dyadic interpersonal synchrony, see (40)]. Different algorithms for calculating synchrony have been used, from the concordance index (24) to windowed cross-lagged correlations (18) and recurrence quantification analysis (41). Often, studies are simultaneously trying out a specific algorithm and studying how the embodied variable (such as EDA synchrony) is related to a specific phenomenon (such as the therapeutic alliance or empathy). The same method can also be used in different contexts; windowed cross-lagged correlations (42) have been used in individual psychotherapy (15) and in couple therapy (23). To summarize, we are still making “fishing expedition research,” that is, attempting to discover new information and generate new knowledge. In the long run, the aim is to come to a common understanding of which features are relevant to extract, and to understand how information from the different embodied variables could be integrated. Hopefully, this will lead to a common theory of the importance of embodied aspects in clinical interactions. Some theoretical suggestions have been developed on, for instance, the relationship between movement synchrony and the therapeutic alliance by Koole and Tschacher (43). They suggested that synchrony is related to this therapeutic alliance by enabling access to another's internal states via interbrain coupling, which over time may improve emotional regulation and thus the therapy outcome. The difficulty is that the theoretical model has not yet been tested empirically.

During the RM project, we learned that the design was feasible and useful in detailing the roles of body language and interpersonal synchrony as healing mechanisms of couple therapy. Even though research on synchrony in a multiperson context, such as couple therapy, is more complex than in dyadic contexts, we found that statistically significant interpersonal synchrony in sympathetic activity (20) and movement synchrony (23) occurs during couple therapy sessions. In successful therapies, sympathetic synchrony increased between the spouses toward the end of the therapy (25), whereas movement synchrony was related to the spouses' well-being and the therapeutic alliance (23). We developed computational tools for analyzing and visualizing the peaks and synchronies in psychophysiological signals and integrated these into qualitative analyses of conversations (10, 13, 32, 44).

**Figure 1:**
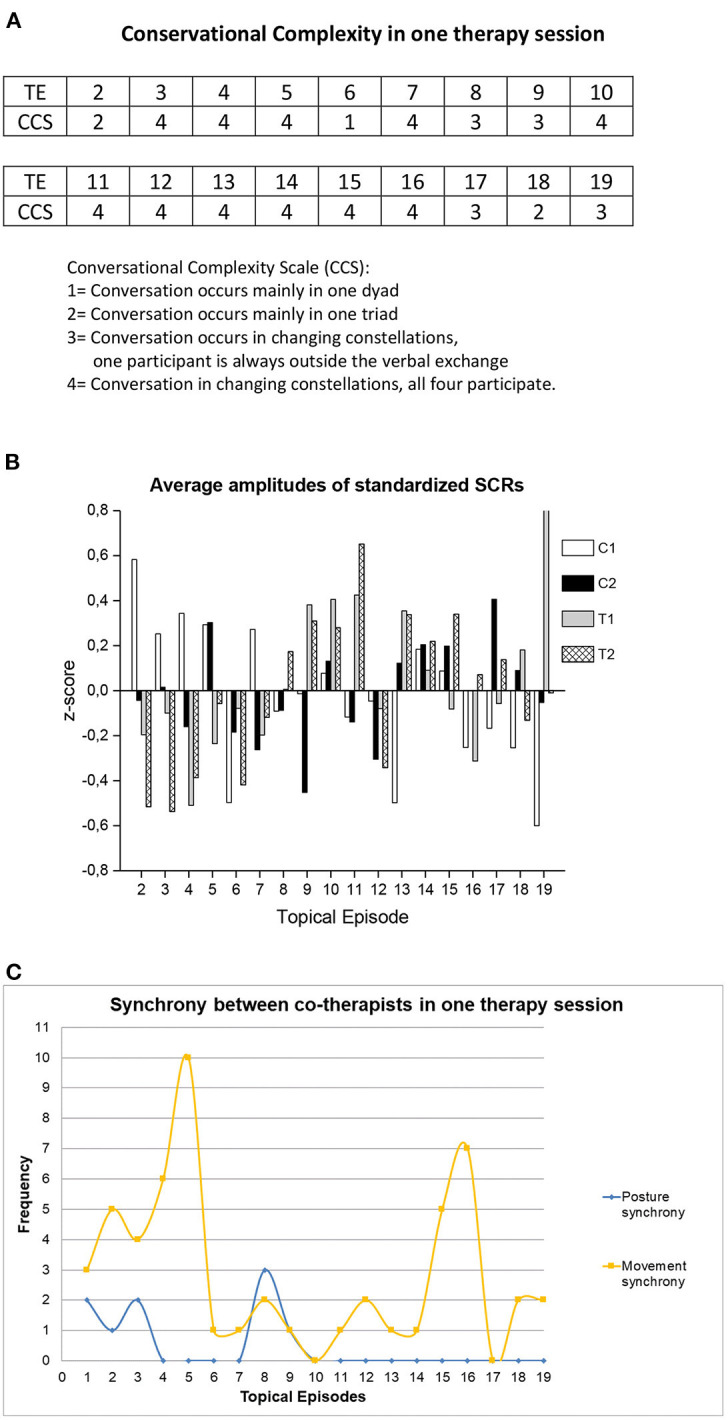
Visualizations of different variables from one couple therapy session. **(A)** For each topical episode (TE) Conversational complexity (CCS) was observed and rated. **(B)** The average arousal levels as standardized Skin Conductance Responses (SCRs); measured = represented = indexed for each topical episode. C1 = client 1, C2 = client 2, T1 = therapist 1, T2 = therapist 2. The panel **(B)** has previously been published in (45). **(C)** The frequency of the co-therapists' posture and movement synchrony per topical episode. **(A)** presents coding of Conversational Complexity for shorter thematic segments (Topical Episodes), and **(B,C)** visualize the embodied variables per topical episode.

**Figure 2:**
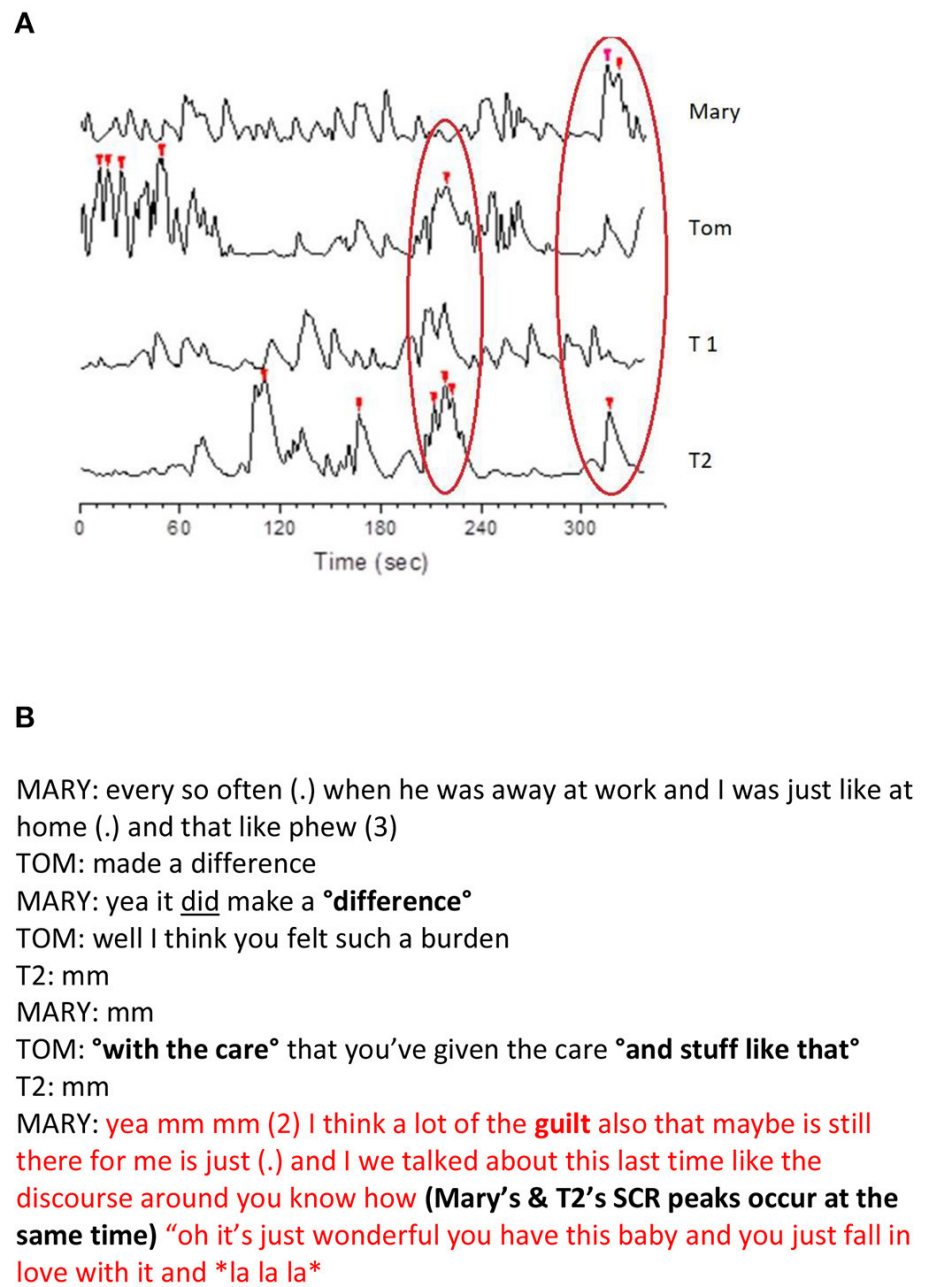
Visualization of the embodied recordings and a transcript of the dialogue during the SCR peaks. **(A)** The synchronization of the participants' SCR peaks during a short segment of dialogue. The peaks marked with red triangles are the SCRs that were >2 SD of the participant's mean. The left red oval depicts synchrony between Tom and therapist 2 and the right oval depicts synchrony between Mary and therapist 2. **(B)** The dialogue occurring at the moment of Mary's and therapist 2's highest SCR peaks (right red oval in **A**). Both panels present a microanalytic study on moments in couple therapy during which there were several SCR peaks between participants. The embodied variables were used together with the transcript of the moment. T1, therapist 1; T2, therapist 2.

For examples of how the embodied variables can be visualized for use in subsequent analyses, see Figures 1, 2. Figure 1 presents analyses on a session level, and Figure 2 presents a microanalytic study of the dialogue. In Figure 1A, the conversational structure between four participants demonstrates that it may vary in different topical episodes (which are episodes of the dialogue that have been separated manually by their thematic contents); verbal exchange may occur only in one dyad, in a triad, in rapidly changing dyads and triads, and also in the quartet. In couple therapy with two therapists, it is common for one or two participants to be outside the verbal exchange for several minutes time until the conversational structure changes (13). Figures 1B,C represent visualizations of each participants' skin conductance responses (panel B) and observed posture and movement synchrony of the co-therapists (panel C) in the topical episodes of one couple therapy session. The information from these pictures was used together with information from the other modalities to analyze the couple therapy session, integrating embodied variables and the dialogue. Figures 2A,B present how we used the embodied variables in relation to the verbal dialogue. Synchrony of the SCR peaks was found between the two dyads in this particular extract. During the RM research process, we discovered important aspects that the researcher must consider when studying embodied variables; we will review them next.

## Gathering and processing the embodied data

One of the most important aspects when gathering data simultaneously from different modalities is that the data are synchronized in a timely manner [see, for instance (46), who describe in detail the choices that the researchers made during the development of a study using wearable equipment to record multimodal data]. Unfortunately, data are still largely synchronized manually; furthermore, there is a lack of providers that enable gathering data from several modalities simultaneously and automatically providing synchronized data. Currently, wearable trackers are still in a developmental phase, but in the future, we hope to see systems that would be easy to use outside laboratory settings in ecologically valid situations, such as psychotherapy clinics. We further hope that the systems would, as stated previously, enable gathering data from different modalities simultaneously within the same system and that the data would be synchronized automatically.

One important aspect to consider when gathering data is its storage. As we have studied clinical material, such as psychotherapy sessions, there are ethical considerations that need to be taken into account in the storage of the data. Many machine learning and deep learning algorithms, which could be suited for analyzing embodied data, use artificial intelligence-based on cloud-based storage. It is possible to store unidentifiable and anonymous data in clouds, but as we have studied the embodied aspects of psychotherapy, we often use video recordings of the actual sessions in our analyses. Even though new ethical guidelines for using artificial intelligence have been developed (47), we need a thorough discussion in the scientific community about how confidential and highly sensitive material, such as psychotherapy videos, which cannot be anonymized, could be used in machine learning applications.

During the research process, we understood that one of the core aspects when studying embodied variables was to consider the properties of the variables of each specific modality separately to thoroughly understand how the data are usually processed and how this affects the information that can be obtained from that modality. For instance, EDA is one of the oldest psychophysiological measures for recording the activity of the autonomic nervous system and its sympathetic nervous system activity. Sympathetic nervous system activity prepares the body for action and is also related to emotions, motivations, attention, and stress (48). EDA is most commonly represented by skin conductance responses (SCR), which are phasic responses to specific stimuli, whereas the skin conductance level (SCL) represents a tonic or slowly changing level of EDA. We used SCRs because of our interest in looking at the participants' reactions in relation to a particular stimulus, namely to what happened in the psychotherapy session (33, 44, 45). The temporal properties of SCR are such that the response happens 1 s after the stimulus and lasts for ~2 s. This is important to consider when analyzing the data. SCRs are temporally exact and thus suitable to use in combination with spoken dialogue in real time—as it is happening. Heart rate variability (HRV), on the other hand, is calculated (and extracted) in longer windows of 15 s (18) and sometimes in intervals of 2–5 min (49). This makes HRV a less time-accurate measure. However, one important aspect is that through the processing of the data, temporal exactness can be affected.

For instance, it is quite common to calculate the peak points of the embodied variable responses (see Figure 2A); however, different ways to calculate the peaks exist. We used two different methods to calculate the peaks in different modalities. The absolute stress vectors (ASVs) were calculated based on the heart rate, the high-frequency power, the low-frequency power, and respiratory variables (50). The ASVs were used to identify the most stressful situations for each participant when the dialogues were studied in couple therapy sessions (10, 32). We also used EDA peaks, which were extracted from the SCRs. The SCRs were first standardized for each participant, and peak detection was then applied. Peaks that were two or more standard deviations above the individual's session mean were selected as statistically significant SCRs (44). In the analyses, the peaks were integrated with what happened in the conversation. Päivinen et al. (44) found EDA peaks in most of the participants in interactions during which one spouse's identity was blamed, i.e., who you are as a person.

As there are multiple ways to extract and process data, the researcher needs to understand the process and how it affects what the information obtained could reflect. For instance, ASV and EDA peak detection are both thought to reflect the activation of the sympathetic nervous system, but ASV is calculated based on heart rate and EDA from sweat secretion in the palms. As the gathering and processing of the data from different modalities require different knowledge and understanding of the data, our research group has designated specific researchers to work on specific modalities, thereby enabling them to acquire expertise on the properties of their specific modality.

## Data analysis strategy

When selecting how to analyze the data, it is important to consider the temporal aspects of each modality. Are the specific variable and the way the data have been extracted and processed best suited for an analysis in which the data analysis unit is temporally very precise, such as combining the embodied variable with a microanalytic qualitative analysis of a transcript (13, 44)? Or is the variable and how it is operationalized better suited for studying longer intervals? We have, for instance, also used SCRs in relation to case studies in which the psychotherapy session was split into topical episodes during which one specific topic was discussed (see Figure 1B). Topical episodes could vary in time, ranging from 2.5 to over 7 min (33, 45). In the qualitative analysis of multimodal data, the researcher needs to consider multiple temporalities, referring to the fact that there are several non-linear overlapping sequences of action in the different modalities, as well as multiactivity, where one or more participants engage in multiple activities at the same time (51).

In qualitative studies, the transcripts of conversations are often used as an automatic part of the analysis process. One innovative methodological suggestion on how to use transcripts in a more embodied way was put forward by Chadwick (52), who emphasized the importance of embodiment by altering the way transcripts were written to include rhythm or embodiment. In the qualitative studies, we mainly used the transcripts of the sessions and included the embodied variables in them.

In quantitative studies on synchrony, for instance, the temporal structure most commonly covers the entire therapy session, as the embodied variables usually display the mean of synchrony in the entire therapy session (20, 23, 25); however, discussions of whether to study session-level synchrony or momentary synchrony are emerging.

## The meaning of the embodied responses

Throughout our research in the RM project, we held one key aspect of studying embodied variables in clinical settings to be what hypotheses the researcher formed regarding the meaning of the responses by a specific variable. For instance, EDA arousal has been related to emotional arousal (53), but also to cognitive load or movement (54). In clinical encounters, EDA arousal has been related to situations in which the therapist is empathic toward the client (55), when there are moments of confrontation (56), when one's identity is blamed (44), and in relation to reflection (45). In a case study, ASV was observed both in the client and in the therapist at an important moment of change, which was thought to relate to both the emotional importance and the psychological importance of the moment (32). In the individual SRIs, both the client and the therapist confirmed the importance of this moment to the client's therapy. This finding is in line with del Piccolo and Finset's (6) suggestion that arousal could be a sign of active emotional engagement. More than anything else, these studies demonstrate the creative exploratory phase of studying embodied variables in clinical settings. We do not yet have the answers to questions such as what EDA or HRV are related to in the clinical context but are slowly accumulating study results. We hope that future research will develop a theory on how arousal is related to the therapeutic process and therapy outcome.

It is also important to form a hypothesis about how context influences embodied responses. Psychotherapy is a specific context that has its own conventions that differ from ordinary interactions. This affects the interpretation of the embodied responses [cf. (57)]. Some of the important aspects that can affect the embodied variables are the behavior and overt responses of all participants that can be related to the topics discussed. At the same time, however, the implicit level is always present. The participants might not be aware of this, but it might still affect the embodied variables. In the RM research project, we used individual interviews (SRIs) to provide additional information about the participants' experiences of the interaction. The SRI can provide access to participants' thoughts, feelings, and bodily sensations during the therapy interactions, but also invite new reflections and insight (58). One instance in which the therapist's feelings were present in the embodied patterns occurred when one of the therapists reported in the SRI that he had felt empathetic toward the client (whereas the other therapist did not), and in the session, he was repeatedly imitating the client's postures and movements (13). In the SRI, participants sometimes commented on their bodily responses and/or emotional behaviors while watching the video clips from the session, e.g., “*Now I'm holding my breath*,” or “*I did not notice myself feeling bitterness during the session, but when I see and hear myself, this looks and sounds like bitterness*,” or “*I was trying not to weep; I often try to hide my feelings*.”

However, in the context of psychotherapy, one of the most important aspects is the relationship between the participants. Synchrony studies have focused on this aspect, but the relationship might also influence individual embodied variables. For instance, relational tension between therapist and client has been reported as physiological arousal (59). There is also the possibility that a defense mechanism, such as suppression, affects the expression of the emotion, but the embodied responses might remain intact (60). It is a complex task to interpret the causes of the embodied responses based on the video material alone. Along with the SRI, these issues can be addressed systematically using self-ratings.

## Methods for calculating synchrony

Our quantitative studies on RM have focused on interpersonal synchrony in the sympathetic nervous system (20, 25) and movements and postures [by two separate methods: (23, 61)]. As the synchrony of a certain embodied variable is studied, this brings forth other important aspects. As previously mentioned, practices on how to calculate synchrony differ from one modality to another, but also within one modality, several methods have been used. The choice of algorithm is often guided by common practice in previous studies of the specific modality. For instance, EDA synchrony has been calculated by the concordance index (18, 20, 21, 24, 25), but also using the windowed cross-lagged correlations (WCLC) (18). Movement synchrony is usually calculated using WCLC (15), but specifics on how the time series are divided into smaller segments (non-overlapping or overlapping windows) and how synchrony is calculated within them also vary. In synchrony algorithms, either z-transformed correlations or concordance indexes have been used as an index of synchrony (62). The statistical significance of the synchrony is determined by shuffling methods using either individual client–therapist or client–client pairs or using a pool of all pairs included in the study (63).

Comparisons of the different methods are scarce. Tschacher and Meier (18) compared the concordance index and the WCLC on EDA synchrony data and found differences between the results obtained by different algorithms. Schoenherr et al. (64) compared the different algorithms used to calculate movement synchrony and demonstrated that different algorithms yielded different results and thus portrayed different kinds of synchrony. Schoenherr et al.'s (64) article is the first methodological study to aim at understanding how the differently calculated synchronies convey different aspects of the same embodied variable. Recently, Altmann et al. (65) compared different algorithms and recommended standardization efforts for synchrony computations. We find that it is important, and sometimes required, for a researcher to understand all the finesses of calculating synchrony; it is vital to thoroughly understand what the calculated synchrony is for the researcher to be able to make valid interpretations of the results.

With regard to understanding synchrony calculations, another fascinating aspect of studying synchrony is that, even though it has been studied a great deal, little is known about the function synchrony has in psychotherapy. In our view, it is important that the researcher forms a hypothesis about what the synchrony of certain embodied responses is thought to reflect. Studies with larger data sets have proven that movement synchrony is related to therapeutic alliance and outcome in individual psychotherapy (15, 16). However, as research has accumulated, a more diverse picture emerges. High movement synchrony has been related to non-improving clients dropping out of therapy (66) as well as better outcomes and faster improvements for clients suffering from interpersonal difficulties (67), therapy sessions during which there was not much improvement (68), and confrontational alliance ruptures (69).

The research on physiological synchrony has also been seen as scattered (70). Sympathetic nervous system arousal and EDA synchrony has been related to empathy between therapist and client (21, 24, 37). Palumbo et al. (70) list the main problems in the research field related to inconsistency in terminology and physiological variables used, as well as methodological and statistical inconsistencies. The same applies to movement synchrony, although there is no existing review on the subject. In the research field, there is still an underlying assumption that more synchrony is better, even though research has repeatedly reported that this is not the case. Mayo and Gordon (71) initiated a discussion on the importance of considering two separate tendencies of synchrony: to synchronize or adapt to the other and to desynchronize or act independently.

As we have studied synchrony in the context of couple therapy, it has become evident that the meaning of synchrony differs if it occurs between spouses, between co-therapists, or between client–therapist dyads [cf. (23, 25)]. In couple therapy, the assumption that more synchrony is always better might not apply, either. In mother–infant studies, too much synchrony has been related to attachment insecurity (72, 73), and between spouses to relationship dissatisfaction and conflict (74). In couple therapy, too much synchrony between spouses could reflect an enmeshment between the spouses—a lack of individuality, leading to dissatisfaction. Another study revealing the complexity of synchrony in couple therapy was a finding that decreased physiological synchrony between therapist and client in a case study was related to a positive therapy outcome (75). For movement synchrony, a similar finding was that too much synchrony led to a reduction in emotional regulation (76).

All studies point to synchrony as a multifaceted phenomenon; differences based on roles have been shown in RM. Another aspect was put forward by Butler (77), who pointed out that synchrony can be seen as something occurring between two persons or as two persons being synchronized to an outside event. As we studied synchrony in couple therapy, we discovered that the therapists were mostly synchronized in both the sympathetic nervous system (20) and their movements (23). This could possibly reflect the two therapists being synchronized to an “outside event,” the spouses' narratives, which are the focus of the therapy.

## Integration of information from different modalities

From the beginning of the RM project, we aimed to integrate information from the various modalities to form a more complete picture of the embodied variables in the therapeutic process. We have systematically developed ways to conduct multimethod, multimodal studies to fully utilize the possibilities offered by this rich and unique data. In multimodal research, several problems need to be solved. Since modality-specific measures operate on different time scales, observations from a single variable can have several meanings, each of which may be relevant to the interaction to a greater or lesser degree. We have tackled these problems with two main strategies: (a) through multimodal case studies, and (b) by using statistical modeling to analyze synchronies in more than one modality simultaneously.

In qualitative case studies, we aimed to integrate information from separate modalities. This was evident from our first study [(10), p. 713], in which we concluded, “*It is evident that it is not enough to look only at the ANS information, or at any other single source of data. We need to integrate … all measured information if we are to make more precise hypotheses and observations on the ways in which the therapist and the client synchronize their embodied reactions in dialog*.” In a microanalytic study on alliance formations in couple therapy (13), which concentrated on an extract of dialogue, we included posture and movement synchrony and EDA peaks in the analysis. The study demonstrated that the different modalities have separate but complementary tasks in couple therapy. When there were clear markers of alliance in a dyad in verbal exchange, there were also markers of synchrony in one or more nonverbal modalities. In addition, when a dyad was outside conversational exchange, markers of nonverbal alliance in the form of bodily synchrony were often observed in the same dyad. In another case study, we combined the dialogue and the average SCR arousal, and posture and movement synchrony to clarify the variables' relationships to each other within the couple therapy session (33). We discovered that the different modalities often told different stories of the same important moment. The naïve assumption, that one modality would represent one aspect of interaction (for instance, EDA arousal reflecting emotions or movement synchrony reflecting rapport) did not receive support. It seems that the responses from the different embodied modalities were highly related to both the context (what was talked about), the emotions, and the participants' individual thoughts, responses, and even defenses.

Qualitative case studies reveal that couple therapy sessions are temporally interesting and vary in unpredictable patterns concerning the dialogue, who talks and who listens, and the embodied variables. These studies questioned the use of session-level variables of embodied variables. Yet, quantitative studies on embodied aspects in psychotherapy often use session-level averages to describe, for instance, synchrony between participants.

Quantitative studies on the relationship between synchronies in different modalities are almost non-existent. In a recent study, we investigated how physiological synchrony, movement synchrony, and speech patterns were associated in couple therapy (78). EDA synchrony and movement synchrony correlated with each other on the entire sample level, especially in the client–therapist dyad. This supports the assumption that the role of the participant can influence synchrony patterns. Contrary to our hypothesis, movement synchrony was negatively correlated with the amount of speech. This study is one of the first attempts to clarify the relationship between synchrony in different modalities and in relation to speech, which is often seen as the key ingredient of therapy.

In the exploratory phase, new methods are created for solving existing methodological problems, such as the new multivariate form of a synchrony algorithm [mv-SUSY (79)], which enables calculating synchrony between multiple participants. Until now, synchrony has been studied mainly in dyadic constellations, even though couple therapy provides an opportunity to study triadic or quadratic synchrony (61). It remains to be seen how this will change studies on synchrony and what new information it will bring about.

One of the aspects that a researcher encounters when studying embodied variables is the mind-body issue, which we have not addressed in RM. As we have studied the multimodality of interaction, it has become apparent that we make inferences of what occurs in the mind of the participant based on the information from the embodied variables. But it is evident that reducing the mind into a simplistic model is not making justice to the complexity of the human mind. The question of how the responses of the body (the embodied variables) are connected to the experiences and processes in the human mind is a classical philosophical question for any researcher in empirical psychology. In the RM, we have tried to approach this dilemma by using the SRIs, wherein we have asked the participants to recall their embodied responses and explicate them to the researcher [cf. (33)]. This methodological endeavor does not, however, answer the question of how the mind and body are connected, for which multi-disciplinary collaboration with for instance philosophers would be both critical and fruitful.

## The added values of using embodied variables

Thus far, we have mainly addressed the challenges of studying the embodied aspects of psychotherapy. Next, we would like to discuss our notions of what the added values are. One of the main advantages is that it enables a new embodied aspect of the interaction to become “visible” and thus increases awareness of the impact the embodied variables have on the clinical interaction. The importance of considering the embodied aspect of the interaction during the psychotherapeutic encounter has been stressed (1, 4, 80–82), since by concentrating solely on the verbal discourse, a narrow view of psychotherapy is constructed. It is our view that taking the embodied variables into account broadens our understanding of the process of change in psychotherapy since our assumption is that a notable part of the change occurs through the embodied connection between therapist(s) and client(s). Actually, this is not a new idea in psychotherapy since the psychoanalytic school of psychotherapy emphasizes making the implicit realm more explicit by giving words to our (embodied) experiences. It is important that some aspects of interactions are most commonly expressed only nonverbally, such as crying/weeping as a sign of sorrow or engagement in the interaction by gazing intently at one's interaction partner.

Our research team has noted that including the embodied side has expanded the view of who benefits from therapy. By observing the interactions in our data set, we noticed that while male clients are sometimes unable to verbalize their experiences, they convey much information through the embodied channels. Hopefully, there will be more embodied ways of evaluating outcomes for psychotherapy in the future. Marci and Riess (83) suggested that studying psychophysiology in psychotherapy (as a marker of empathy) could serve as a potential bridge between psychotherapy research and the theory and practice of psychotherapy, which was also suggested over 60 years ago (84).

We would also like to put forth an interesting development in the feasibility of using embodied data in psychotherapy sessions. Including biofeedback in psychotherapy is not a new concept; it has been around for at least 50 years [cf. (85)]. As embodied trackers have become available and affordable to the average client, they offer new opportunities for psychotherapists to include embodied variables in the psychotherapy process. Information from the embodied trackers can be seen as a more objective form of information that could inform the therapist and client about the progress of therapy or important factors contributing to the client's well-being. In her psychotherapy practice, the first author has used information from her client's well-being trackers of sleep quality, HRV, and respiratory rate. The data have revealed important aspects of clients' lives that might otherwise be omitted from awareness and dialogue. Sometimes, clients might not be aware of what stressors they have in their daily lives, and through tracking their embodied variables, they learn to recognize formerly implicit stressors. It goes without saying that it is also interesting for the psychotherapist to wear a tracker and, after each session, to view whether it was a stressful or a relaxing session. Another added value can be seen in using trackers with clients who suffer from psychosomatic disorders. It might be that through discussing the embodied metrics of the client—who is usually very interested in the metrics—the working alliance is slowly developing in a less threatening way. The embodied variables can help in creating the bond between therapist and client. Sometimes, the trackers can reveal important aspects of relational bonding in the embodied metrics, which we haven't symbolized previously. Kleinbub et al. (82) suggested that including biofeedback in psychotherapy could inform important aspects of the interpersonal relationship, which could be used when monitoring the psychotherapy process.

Of course, it is important that the psychotherapist knows what the specific embodied metrics are related to, is curious about using them in their own private practice and is also aware of the benefits and risks of using trackers.

## Recommendations

As studies on the subject already exist, we find it important that researchers familiarize themselves with previous studies so that they do not invent new methodologies if preexisting methods are available. For a researcher considering a broader view of what aspects need to be considered in the research, we refer to Spatz (86). For a more theoretical way of defining research on embodiment, we refer to Brown et al. (87).

We urge more collaboration between researchers to avoid the pitfall of reinventing the wheel. An ongoing dialogue and more effective networking between experts of the different modalities from separate research groups would increase our in-depth understanding of the various modalities and their interrelations in the context of psychotherapy. It is important to create recurring meetings, such as summer schools or international seminars and data seminars, for psychotherapy researchers who include embodied variables in their research. This would help to develop the field and enable us to start making joint decisions on what analysis units to use, which calculation methods for synchrony would be best suited to our research aims, and which parameters to choose. This could, in turn, help make the various studies more comparable, and information would start to accumulate on the different aspects of embodiment in psychotherapy.

Another important development would be to define the context of psychotherapy, to create a model based on the common factors of change in psychotherapy, and to clarify how psychotherapy interaction differs from everyday interactions. Ideally, there would be a dialogue between researchers studying embodied variables in in non-clinical institutional contexts, everyday interactions and psychotherapy researchers. Obtaining information from the principles of how the embodied variables react during interaction would be important information for psychotherapy and basic research.

The same would apply to synchronization studies in the context of embodiment; self-organized meetings of researchers of human interaction with invited experts on engineering, statistics, and computer science could be arranged to develop models for gathering structured data of the experimental and clinical sessions. It would be useful to define, for example, the number of participants, length of sessions, equipment for gathering data on autonomic nervous activity, movements, and speech, protocols for synchronizing the recordings, defining the data structure, and protocols for making the data open access in a safe and ethical manner. In addition, preferred methods for extracting essential features of the data and statistical methods revealing the synchronization of participants based on those features would be defined. Then, strictly defined large datasets would allow the use of deep learning artificial intelligence to dig out the essential features of the bodily, experiential, and cognitive dimensions of the interaction. Furthermore, methods of complexity science could be used to develop, for example, multidimensional complexity models to explain and predict emergent behavioral changes in single person and couple therapy. Based on our research, we would like to suggest that candidate attractor states promoting and/or helping to sustain behavioral change would be, for example, SCR peaks, silent movements in discussion, changes in breathing patterns, and posture changes.

We find that during this phase of the research, it is important to implement both qualitative and quantitative studies, and that researchers in their reports emphasize and reveal their conclusions behind their methodological choices. This would help other researchers develop the research further.

By qualitative studies, the meaning of the underlying embodied variables can be deciphered, and various hypotheses can be developed for further use in quantitative studies. Qualitative studies can aid in developing theories on the relations between embodied variables and various contexts. For qualitative studies, it is important that the embodied variables are not used as proof of bodily influence, but to use them alongside qualitative analyses to explore how the embodied side of the interaction is a constitutive part of social interaction (88).

In quantitative studies, it is vital to further develop the methods, scrutinize the meaning of the differently operationalized embodied variables, and critically evaluate how to interpret the results obtained in different contexts. It is important to develop theoretically informed hypotheses regarding the embodied variables studied and their relationships to other variables. We urge the researcher to question the meaning of the variables and to consider the idiosyncrasy and context dependency of the embodied variables.

It has been suggested that a large part of the success of the clinical encounter has to do with the embodied variables in the interaction (43, 81, 89) and that clinical reasoning by therapists is often grounded in the embodied domain (90). We have discovered that studying embodied variables is vital to understanding how the change process in psychotherapy occurs, and that including embodied variables in the designs enables us to create a more multidimensional and ecologically valid picture of what is truly going on in the psychotherapy process.

## Author contributions

PN-S had the main responsibility of writing and editing the manuscript and figures. V-LK and MP contributed to the writing process and the figures. All authors contributed equally to the substance of the article, revised the article critically for important intellectual content, and approved the submitted version to be published.
