# Supplementary material for: Challenges and added value of measuring embodied variables in psychotherapy
Source: Front Psychiatry. 2022 Dec 16;13:1058507. doi: 10.3389/fpsyt.2022.1058507 (PMC9800897; doi:10.3389/fpsyt.2022.1058507)
Supplement: Supplementary file 1 [file Data_Sheet_1.pdf]

Appendix

Relational Mind publications

Itävuori, S., Korvela, E., Karvonen, A., Penttonen, M., Kaartinen, J., Kykyri, V-L., & Seikkula, J. (2015). The significance of silent moments in creating words for the not-yet-spoken experiences in threat of divorce. *Psychology, 6*, 1360–1372. https://www.doi.org/10.4236/psych.2015.611133

Karvonen, A., Kykyri, V-L., Kaartinen, J., Penttonen, M., & Seikkula, J. (2016). Sympathetic nervous system synchrony in couple therapy. *Journal of Marital and Family Therapy, 42*(3), 383–395. https://doi.org/10.1111/jmft.12152

Karvonen A. (2017). Sympathetic nervous system synchrony between participants of couple therapy (doctoral dissertation). *Jyväskylä Studies in Education, Psychology and Social Research, 599.* https://jyx.jyu.fi/handle/123456789/55925

Kykyri, V-L., Karvonen, A., Nyman-Salonen, P., Kurri, K., Wahlström, J., Kaartinen, J., Penttonen, M., & Seikkula, J. (2019). Alliance Formations in Couple Therapy: A Multimodal and Multimethod Study. *Journal of Couple & Relationship Therapy, 8*(3), 189–222. https://www.doi.org/10.1080/15332691.2018.1551166

Kykyri, V-L., Karvonen, A., Wahlström, J., Kaartinen, J., Penttonen, M., & Seikkula, J. (2017). Soft Prosody and Embodied Attunement in Therapeutic Interaction: A Multimethod Case Study of a Moment of Change. *Journal of Constructivist Psychology, 30*(3), 211–234. https://doi.org/10.1080/10720537.2016.1183538

Laitila, A., Vall, B., Penttonen, M., Kykyri, V-L., Karvonen, A., Tsatsishvili, V., Kaartinen, J., & Seikkula, J. (2018). The added value of studying embodied responses in couple therapy research: A case study. *Family Process, 58*(3), 685–697. https://doi.org/10.1111/famp.12374

Lampinen, E., Karolaakso, T., Karvonen, A., Kaartinen, J., Kykyri, V-L., Seikkula, J., & Penttonen, M. (2017). Electrodermal activity, respiratory sinus arrhythmia, and heart rate variability in a relationship enrichment program. *Mindfulness, 9*(4), 1076–1087. https://doi.org/10.1007/s12671-017-0843-6

Nyman-Salonen, P., Vall, B., Laitila, A., Borcsa, M., Penttonen, M., Tourunen, A., Kykyri, V-L. ... & Seikkula, J. (2020). Significant moments in a couple therapy session: Towards the integration of different modalities of analysis. *In Systemic Research in Individual, Couple, and Family Therapy and Counseling* (pp. 55-73). Springer, Cham.

Nyman-Salonen, P., Tourunen, A., Kykyri, V-L., Penttonen, M., Kaartinen, J., & Seikkula, J. (2021). Studying nonverbal synchrony in couple therapy—observing implicit posture and movement synchrony. *Contemporary Family Therapy, 43*(1), 69–87. https://doi.org/10.1007/s10591-020-09555-5

Nyman-Salonen, P., Kykyri, V-L., Tschacher, W., Muotka, J. S., Tourunen, A., Penttonen, M., & Seikkula, J. (2021). Nonverbal synchrony in couple therapy linked to clients' wellbeing and the therapeutic alliance. *Frontiers in Psychology, 12*:718353. https://10.3389/fpsyg.2021.718353

Paananen, K., Vaununmaa, R., Holma, J., Karvonen, A., Kykyri, V-L., Tsatsishvili, V., Kaartinen, J., Penttonen, M., & Seikkula, J. (2018) Electrodermal activity in couple therapy for intimate partner violence. *Contemporary Family Therapy, 40*(2), 138–152. https://doi.org/10.1007/s10591-017-9442-x

Päivinen, H., Holma, J., Karvonen, A., Kykyri, V-L., Tsatsishvili, V., Kaartinen, J., Penttonen, M., & Seikkula, J. (2016). Affective Arousal During Blaming in Couple Therapy: Combining Analyses of Verbal Discourse and Physiological Responses in Two Case Studies. *Contemporary Family Therapy, 38*(4), 373–384. https://doi.org/10.1007/s10591-016-9393-7

Seikkula, J., Karvonen, A., Kykyri, V-L., Kaartinen, J., & Penttonen, M. (2015). The embodied attunement of therapists and a couple within dialogical psychotherapy: An introduction to the relational mind research project. *Family Process, 54*(4), 703–715. https://doi.org/10.1111/famp.12152

Seikkula, J., Karvonen, A., Kykyri, V-L., Penttonen, M. & Nyman-Salonen, P. (2018). The Relational Mind in Couple Therapy: A Bateson-Inspired View of Human Life as an Embodied Stream. *Family Process, 57*(4), 855–866. https://doi.org/10.1111/famp.12382

Tourunen, A., Kykyri, V-L., Seikkula, J., Kaartinen, J., Tolvanen, A., & Penttonen, M. (2020). Sympathetic nervous system synchrony: An exploratory study of its relationship with the therapeutic alliance and outcome in couple therapy. *Psychotherapy, 57*(2), 160–173. https://doi.org/10.1037/pst0000198

Tourunen, A., Nyman-Salonen, P., Muotka, J., Penttonen, M., Seikkula, J., & Kykyri, V-L. (2022). Associations between sympathetic nervous system synchrony, movement synchrony, and speech in couple therapy. *Frontiers in Psychology, 13*:81835. https://doi.org/10.3389/fpsyg.2022.818356

Vall, B., Laitila, A., Borcsa, M., Kykyri, V-L., Karvonen, A., Kaartinen, J., Penttonen, M., & Seikkula, J. (2018). Stimulated Recall Interviews: How can the research interview contribute to new therapeutic practices? *Revista Argentina de Clinical Psicologica, XXVII*(2), 274–293.
